# Genetic Alterations of NF-κB and Its Regulators: A Rich Platform to Advance Colorectal Cancer Diagnosis and Treatment

**DOI:** 10.3390/ijms25010154

**Published:** 2023-12-21

**Authors:** Faranak Alipourgivi, Aishat Motolani, Alice Y. Qiu, Wenan Qiang, Guang-Yu Yang, Shuibing Chen, Tao Lu

**Affiliations:** 1 Department of Pharmacology & Toxicology, Indiana University School of Medicine, Indianapolis, IN 46202, USA; falipour@iu.edu (F.A.); amotolan@iu.edu (A.M.); 2 Indiana University Melvin and Bren Simon Comprehensive Cancer Center, Indianapolis, IN 46202, USA; 3 Center for Developmental Therapeutics, Chemistry of Life Processes Institute, Northwestern University, Evanston, IL 60208, USA; aliceqiu2023@u.northwestern.edu (A.Y.Q.); w-qiang@northwestern.edu (W.Q.); 4 Department of Obstetrics and Gynecology, Feinberg School of Medicine, Northwestern University, Chicago, IL 60611, USA; 5 Robert H. Lurie Comprehensive Cancer Center of Northwestern University, Chicago, IL 60611, USA; g-yang@northwestern.edu; 6 Department of Pathology, Feinberg School of Medicine, Northwestern University, Chicago, IL 60611, USA; 7 Department of Surgery, Weill Cornell Medicine, 1300 York Avenue, New York, NY 10065, USA; shc2034@med.cornell.edu; 8 Department of Biochemistry & Molecular Biology, Indiana University School of Medicine, Indianapolis, IN 46202, USA; 9 Department of Medical & Molecular Genetics, Indiana University School of Medicine, Indianapolis, IN 46202, USA

**Keywords:** CRC, genetic alteration, NF-κB, ODAD2, organoid, PDX, PRMT5

## Abstract

Colorectal cancer (CRC) is the third leading cause of cancer mortality in the United States, with an estimated 52,000 deaths in 2023. Though significant progress has been made in both diagnosis and treatment of CRC in recent years, genetic heterogeneity of CRC—the culprit for possible CRC relapse and drug resistance, is still an insurmountable challenge. Thus, developing more effective therapeutics to overcome this challenge in new CRC treatment strategies is imperative. Genetic and epigenetic changes are well recognized to be responsible for the stepwise development of CRC malignancy. In this review, we focus on detailed genetic alteration information about the nuclear factor (NF)-κB signaling, including both NF-κB family members, and their regulators, such as protein arginine methyltransferase 5 (PRMT5), and outer dynein arm docking complex subunit 2 (ODAD2, also named armadillo repeat-containing 4, ARMC4), etc., in CRC patients. Moreover, we provide deep insight into different CRC research models, with a particular focus on patient-derived xenografts (PDX) and organoid models, and their potential applications in CRC research. Genetic alterations on NF-κB signaling components are estimated to be more than 50% of the overall genetic changes identified in CRC patients collected by cBioportal for Cancer Genomics; thus, emphasizing its paramount importance in CRC progression. Consequently, various genetic alterations on NF-κB signaling may hold great promise for novel therapeutic development in CRC. Future endeavors may focus on utilizing CRC models (e.g., PDX or organoids, or isogenic human embryonic stem cell (hESC)-derived colonic cells, or human pluripotent stem cells (hPSC)-derived colonic organoids, etc.) to further uncover the underpinning mechanism of these genetic alterations in NF-κB signaling in CRC progression. Moreover, establishing platforms for drug discovery in dishes, and developing Biobanks, etc., may further pave the way for the development of innovative personalized medicine to treat CRC in the future.

## 1. Introduction

### 1.1. Overview of Colorectal Cancer (CRC)

CRC comprises colon and rectal cancers and is one of the most aggressive forms of cancers [1]. According to the American Cancer Society, about 52,000 people are estimated to die from CRC in 2023, making it the third leading cause of cancer mortality in the United States [1]. Notably, considerable advances have been made in the screening and early diagnosis of CRC, resulting in a 60–90% five-year overall survival rate for both local and metastatic CRC patients, particularly for rectosigmoid colon and left colon cancer [2]. Upon diagnosis, patients typically undergo surgical resection and are administered a single or a combination chemotherapeutic regimen, which comprises 5-fluorouracil, oxaliplatin (OX), irinotecan, and capecitabine [3,4]. The challenges are the genetic heterogeneity of CRC which lead to possible relapse and drug resistance. Thus, there is an urgent need to develop more effective therapeutics to overcome the aforementioned challenges in CRC treatment strategies [5].

### 1.2. Model of CRC Initiation and Progression

**Figure 1:**
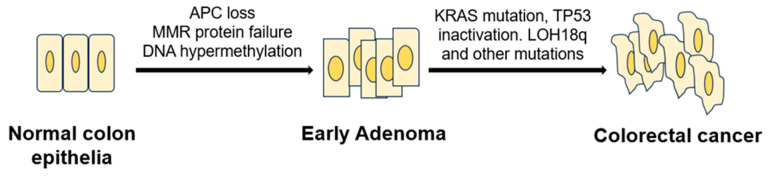
Schematic demonstrating the stepwise accumulation of genetic aberrations that lead to the development of CRC. Sporadic development of CRC occurs when normal colorectal epithelia are transformed into an early adenoma through the silencing of adenomatous polyposis coli (APC), or DNA mismatch repair (MMR) protein failure, and DNA hypermethylation, resulting in the enhanced expression of genes involved in cellular differentiation, growth, and cell cycle. Following the development of a dysplastic colorectal epithelia, the mutation of KRAS and inactivation of TP53, alongside other genetic and epigenetic changes, lead to the development and subsequent progression of CRC.

Genetic and epigenetic alterations underlie the stepwise development of CRC malignancy. The development of CRC begins as an irregular colonic crypt evolving into a polyp, which then accumulates genetic changes to progress into CRC over 10–15 years [6]. The most common pathway is the adenoma–carcinoma sequence. According to the Vogelstein model, sporadic development of CRC occurs when normal colorectal epithelia are transformed into an early adenoma through the silencing of adenomatous polyposis coli (APC), a known negative regulator of the Wnt signaling pathway. The hypoactivity of APC results in the stabilization of β-catenin, allowing the enhanced expression of genes involved in cellular differentiation, growth, and cell cycle [7]. Following the development of a dysplastic/adenomatous colorectal epithelia, the mutation of KRAS and inactivation of TP53, alongside other genetic and epigenetic changes, lead to the development and subsequent progression of a colorectal adenocarcinoma [8]. Another less common pathway for CRC development is the serrated neoplasia pathway, which encompasses the progression of a hyperplastic/sessile serrated polyp to a microsatellite stable/instable (MSS/MSI) carcinoma [9]. Particularly, MSI, which is detected in about 15% of CRC patients, is caused by a deficiency in the activity of DNA mismatch repair (MMR) proteins, such as MSH2, MLH1, PMS2, PMS1, and MSH6 [10]. Some of the MSI patients’ tumors contain mutations that either developed sporadically or are passed down hereditarily (Lynch syndrome CRC). Also, epigenetic instability, through hypermethylation of the CpG Island contributes to CRC tumorigenesis. This subset of CRC, known as the CpG island methylator phenotype (CIMP), is characterized by the silencing effect of methylation at the DNA of several tumor suppressor genes, thus leading to their activation [11] (Figure 1).

### 1.3. Known Genetic Changes in CRC

CRC is often characterized by the changes in its genetic landscape. The classification of CRC based on its predominant molecular pathways includes a chromosomal instability pathway, a microsatellite instability pathway, and a CpG island methylator phenotype. These CRC subtypes are associated with alterations in a specific set of tumor suppressors and oncogenes [12]. For example, as aforementioned, the APC gene mutation is a well-established driver of CRC initiation. The inactivation of APC enhances the activity of cyclin D1, c-myc, c-Jun, which promotes cellular proliferation [13]. Furthermore, mutant KRAS, found in 30–50% of CRC tumors, influences the activity of the RAF/MEK/ERK and PI3K/PTEN/AKT signaling pathways, leading to cascade of events that fuel worse overall survival [8]. For example, the aberrant activation of the RAF/MEK/ERK pathway by mutant KRAS leads to the upregulation of insulin-like growth factor-I receptor (IGF-IR), thus promoting CRC metastasis to the liver [14]. KRAS mutation also impacts normal cellular polarity, protein synthesis, angiogenesis, and cellular death functions in CRC cell lines and patients [15]. Notably, the mutation of valine (V) to glutamic acid (E) at residue 600 on BRAF (BRAFV600E), a kinase downstream of KRAS, is found in microsatellite stable CRC patients and contributes to poor clinical outcomes, including the resistance to anti-epidermal growth factor receptor (EGFR) therapy [16]. Somatic mutations of phosphatidylinositol 3-kinase (PI3KCA) are found in ~30% of CRC and encompass the loss of PTEN, and the amplification of insulin receptor substrate 2 (IRS2), AKT, and PAK4 [17]. Similarly, the mutation and loss of TP53 are reported in up to 75% of CRC cases. TP53 loss compromises the cell cycle regulation in CRC through the suppression of BubR1, p21, and p27 activities [7]. Also, the loss of heterozygosity at the long arm of chromosome 13 (18q LOH) is found in 80% of CRC patients [18]. Some of the key tumor suppressor genes that are affected by this chromosomal aberration include small mothers against decapentaplegic homolog (SMAD) 2, SMAD4, netrin receptor DCC (DCC), and Cdk5. Notably, SMAD2 and 4 are terminal effectors of the transforming growth factor (TGF)-β signaling pathway, and they play a role in suppressing protein translation, cellular growth, and proliferation, and epithelial to mesenchymal transition (EMT) [18]. Collectively, there are several other genetic alterations that drive CRC development and progression, and some others remain to be uncovered. Subsequently, through in-depth sequencing strategies, scientists will be able to better stratify CRC patients genetically to provide tailored effective therapeutics [19,20].

## 2. NF-κB Signaling in CRC

**Figure 2:**
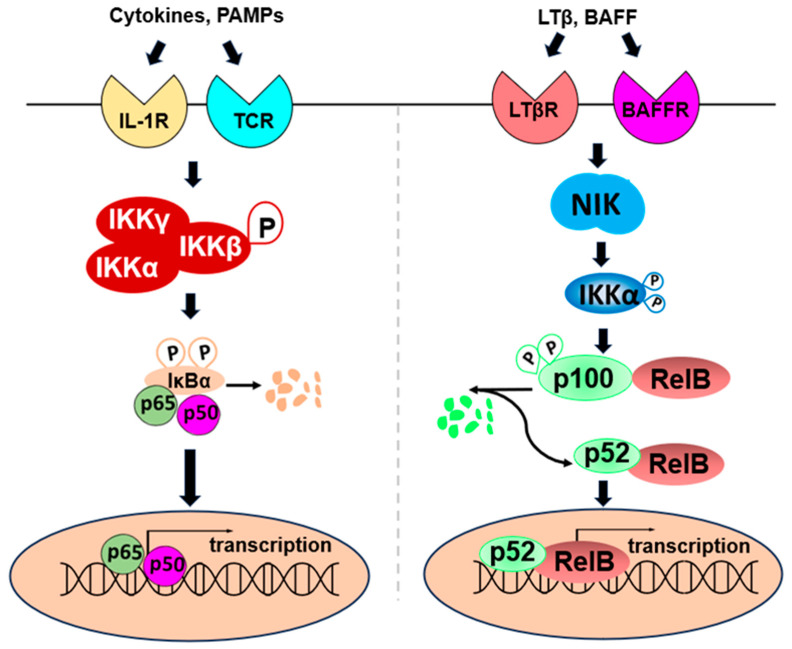
Schematic illustration of simplified NF-κB pathways. (**Left**): Canonical pathway: Upon stimulation by various factors, like cytokines or pathogen-associated molecular pattern molecules (PAMPs), IKK complex will be activated, leading to the phosphorylation of IκBα and its subsequent degradation. This will free up the p65/p50 heterodimer, which will then translocate into the nucleus, bind to κB-binding site, and trigger the transcription of NF-κB dependent genes. (**Right**): Non-canonical pathway. Upon stimulation by various factors, like lymphotoxin (LT) β or B lymphocyte activating factor of the tumor necrosis factor family (BAFF), NF-κB-inducing kinase (NIK) will be activated, leading to the phosphorylation and activation of IKKα, and the subsequent processing of p100 (p52 precursor)/RelB into mature p52/RelB heterodimer. This p52/RelB heterodimer will then translocate into the nucleus, bind to the κB-binding site, and trigger the transcription of NF-κB dependent genes. Symbol P: phosphorylation.

NF-κB is a master regulator of inflammation and plays a critical role in triggering the expression of tumor-promoting genes [21]. NF-κB comprises five transcription factors—p65 (RelA), RelB, c-Rel (Rel), NF-κB1 (p50/p105), NF-κB2 (p52/p100)—which dimerize and bind to gene promoters to facilitate NF-κB transcriptional activity. Other major components of the NF-κB signaling include a multi-subunit IκB kinase (IKKα, β, and γ) and the inhibitors of NF-κB (IκBα, IκBβ, and IκBε) [22]. Based on the sets of stimuli received and the series of signaling cascades that result intracellularly, the NF-κB signaling pathway is divided into two pathways: canonical and non-canonical pathways. In the canonical pathway, proinflammatory signals, such as cytokines, growth factors, pathogen-associated molecular patterns (PAMPs), etc., lead to the phosphorylation of IKKβ, thus causing its activation. The activated IKKβ further phosphorylates IκBα, resulting in its proteasomal degradation. The degradation of IκBα exposes the nuclear localization motif on NF-κB, leading to its nuclear translocation and subsequent induction of gene transcription [23]. NF-κB’s primary effectors in the canonical pathway include p65/p65, c-Rel/c-Rel, p65/p50, and c-Rel/p50 [24]. Together, their activities affect key cellular functions such as inflammation, cell survival, and cell death [25]. On the other hand, non-canonical NF-κB signaling involves the activation of receptors such as the cluster of differentiation 40 (CD40) receptor, the lymphotoxin-β receptor (LTβR), and BLyS receptor 3 (BR3) receptors, which then activate NF-κB inducing kinase (NIK) and IKKα. Activated IKKα phosphorylates p100, causing partial proteasomal degradation to produce p52, which predominantly associates with RelB and translocates into the nucleus to bind to their cognate genes and promote transcription [26]. Quite different from the canonical pathway, the majority of the genes activated by this non-canonical pathway are involved in biological functions like lymphoid organogenesis, B-cell survival and maturation, dendritic cell activation, and bone metabolism [27] (Figure 2).

Notably, the canonical NF-κB signaling pathway has been highly implicated in CRC tumorigenesis as evident from its aberrantly hyperactivated signaling in ~50% of CRC patients [28]. Ping and colleagues reported that IL-1β activated NF-κB signaling promotes CRC growth via increased expression in miR-181a, which negatively regulates PTEN expression [29]. Several studies have also demonstrated the critical role of NF-κB in CRC cell proliferation, anti-apoptosis, inflammation, metastasis, and therapeutic resistance via upregulation of oncogenes that drive those biological functions [30]. As NF-κB is an important node of signaling that drives CRC, it is important to dissect how mutations and other genetic/epigenetic aberrations directly influence NF-κB signaling components and their activity in CRC.

## 3. Genetic Changes of NF-κB Signaling in CRC

Both NF-κB family members and the regulators of the NF-κB signaling pathway have been identified to harbor mutations in CRC patients. Using a large group of 348 CRC patients’ samples from CBioportal [31,32], we will summarize some protein mutations in NF-κB and its regulators in CRC below.

### 3.1. Known NF-κB Genetic Mutations in CRC

#### 3.1.1. Genetic Alterations

As summarized in Table 1 [31,32], different components of NF-κB family members have various gene alterations, like mutations, deletion, amplification, etc., in CRC.

**Figure 3:**
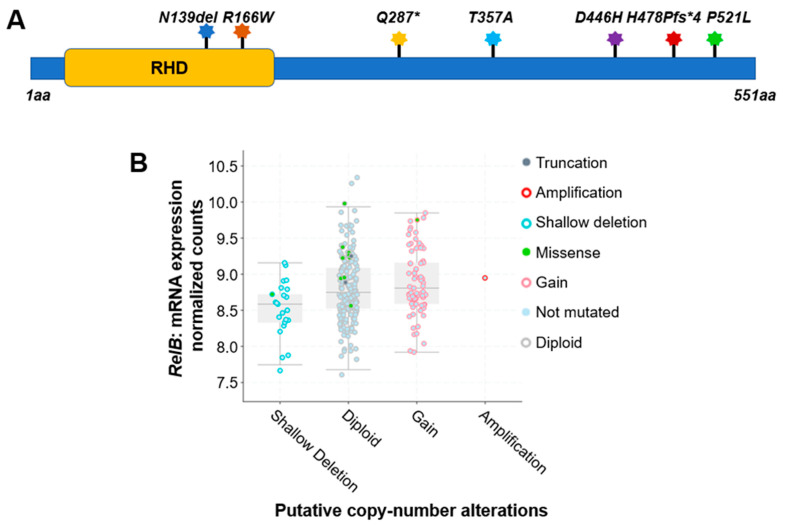
Genetic alterations on NF-κB family members RelA and RelB. (**A**) Schematic diagram of protein mutations on RelA identified in CRC patients. Data resource: cBioPortal for Cancer Genomics [31,32]. Note: RelA is 551 amino acids (aa) in length. Abbreviation: RHD: Rel homology domain. (**B**) Putative copy number alterations from Genomic Identification of Significant Targets in Cancer (GISTIC) for RelB. Data resource: cBioPortal for Cancer Genomics [31,32]. Symbol *: Nonsense mutations.

For instance, a comprehensive analysis of 348 colon cancer samples revealed that approximately 2.5% of the cases exhibited gene alterations in the RelA protein. As shown in Table 1 and Figure 3A, seven different types of protein mutations have been identified on RelA, the large subunit of NF-κB, in 348 CRC patients. They are *R166W* (Arginine–Tryptophan mutation), *D446H* (Aspartic acid–Histidine mutation), *N139del* (Asparagine deletion), etc. These mutations include missense, deletion, insertion, or nonsense mutations. The copy number of gene is either diploid, or shallow deletion. Among these mutations, two are located in the rel homology domain (RHD) (Figure 3A), which mediates the crucial function of DNA contact and homo- and heterodimerization.

Interestingly, compared to the RelA protein, another NF-κB family member, RelB, exhibited more frequency of gene alterations. The analysis of 348 colon cancer samples revealed that approximately 5% of the cases exhibited gene alteration in the RelB protein. As shown in Table 1, more than a dozen various types of protein mutations have been identified on RelB in 348 CRC patients. They are *P314L* (Proline–Leucine mutation), *T494M* (Threonine–Methionine mutation), Y539H (Tyrosine–Histidine mutation), etc. These mutations include missense, deletion, or nonsense mutations. The copy number of gene is diploid, gain, shallow deletion, or amplification (Table 1, Figure 3B).

Other NF-κB family members, like Rel, NF-κB1, and NF-κB2, have been identified with various types of gene alterations. The overall mutation types identified among important NF-κB signaling components are listed in Table 1, Table 2 and Table 3 (Data resource: cBioPortal for Cancer Genomics) [31,32]. For instance, the protein mutations types identified for Rel, RelA, RelB, NF-κB1, and NF-κB2 are 5, 7, 13, 4, and 9, with a total number of 38 protein mutation variants identified (Table 3). The gene alteration frequencies are in the same order, 2.5, 2.5, 5.0, 1.8, and 3.0%, respectively, with a total 14.8% alteration frequency among these 5 NF-κB family members. These data suggest the high genetic alteration rate of NF-κB family members in CRC patients, highlighting the genetic heterogeneity of NF-κB family members alterations in CRC, and suggesting potential implications for the proteins’ functional activities in CRC.

#### 3.1.2. Polymorphism in the NF-κB1 Gene in CRC

In addition to protein mutations among NF-κB family members, other genetic changes, such as polymorphism have also been linked to CRC progression. Despite the fact that NF-κB can form various pairs of molecules, the prototypical one is the heterodimer of p65 (RelA)/p50. p50 is coded by the NF-κB1 gene on chromosome 4q23-q24. This p65/p50 heterodimer plays a key role in NF-κB function [33,34,35]. In several cancer types, including CRC, lung cancer, blood cancer, and pancreatic cancer, etc., researchers have observed constitutive activation of this particular family [36,37,38,39].

Importantly, a recent study has detected a genetic variation within the promoter segment of the *NF-κB1* gene. This genetic variation entails a 4-base pair (bp) insertion/deletion (94ins/delATTG) positioned between two presumed critical regulatory components in the promoter, specifically AP-1 and κB [36]. Interestingly, the research found that people who have two copies of this deletion (94del/delATTG) are more likely to develop ulcerative colitis, a chronic inflammatory bowel disease predominantly affecting the colon. If left untreated, ulcerative colitis-related inflammation in the colon can result in an increased risk in developing CRC. The study observed that the deletion variant of the 94ins/delATTG polymorphism in the *NF-κB1* gene’s promoter region, whether homozygous (DD) or heterozygous (WD), was linked to an increased risk in CRC among Swedish patients, including those with both unselected and sporadic forms of the disease [36,40].

Taken together, numerous evidences have demonstrated the high mutation rate of NF-κB family members in CRC. Further investigation is warranted to elucidate the specific biological significance and clinical implications of these diverse NF-κB mutations and mRNA expression patterns in the context of CRC progression and treatment response. Such insights may provide valuable avenues for targeted therapies and personalized approaches in managing CRC patients with these specific molecular features.

### 3.2. Known Genetic Changes of NF-κB Signaling Regulators

#### 3.2.1. Overall Genetic Alterations

In addition to the NF-κB family members mentioned above, there are many known NF-κB signaling pathway regulators that may harbor genetic alterations in CRC patients as well. It is worth noting that both negative regulators of NF-κB subject to loss-of-function mutations or positive regulators of NF-κB affected by gain-of-function mutations may lead to NF-κB constitutive activation.

As shown in Table 2, representative positive regulators such as Inhibitor of nuclear factor κB kinase subunit α (*Chuk*, also named *IKBKA*), Inhibitor of nuclear factor κB kinase subunit β (*IKBKB*), Interleukin 1 receptor associated kinase 1 (*IRAK1*), Mitogen-activated protein kinase kinase kinase 7 (*MAP3K7*), Protein arginine methyltransferase 5 (*PRMT5*) [34,41,42,43], TGFβ activated kinase (*MAP3K7*) binding protein 1 (*TAB1*), *TAB2*, and Tumor necrosis factor receptor associated factor 2 (*TRAF2*) have 5, 7, 7, 9, 6, 4, 5, and 5 different types of protein mutation variants, respectively. In the same order, their individual genetic alteration frequencies are 1.4, 6.0, 4.0, 4.0, 5.0, 1.8, 3.0, and 2.8% (Table 2 and Table 3) (Data resource: cBioPortal for Cancer Genomics) [31,32], with *IKBKB* and *PRMT5* having the top 2 gene alteration frequency.

In terms of negative regulators, such as Lysine demethylase 2A (*KDM2A*, also named F-box and leucine-rich repeat 11, *FBXL11*) [35,39], NF-κB inhibitor α (*NFKBIA*, also named *IκBα*), and Outer dynein arm docking complex subunit 2 (*ODAD2*, also named armadillo repeat-containing 4, *ARMC4*) [44], they have 8, 2, and 17 different types of mutant variants. Accordingly, their genetic alteration frequencies are 2.8, 1.1, and 7.0%, among which *ODAD2* has the most diverse mutants and highest genetic alteration frequency (Table 2 and Table 3).

Together, there are total 75 protein mutation types and 38.9% of genetic alteration frequency in these representative NF-κB positive and negative regulators. With additional 38 mutation types, and 14.8% genetic alteration frequency of NF-κB family members, it consists of a strikingly high number of mutation types (113 types) and high frequency (~53.7%) of genetic alterations in these 348 CRC patients studied (Table 3). In addition to the representative NF-κB regulators listed in Table 2, there are more NF-κB pathway regulators that are not included in Table 2, like myeloid differentiation primary response 88 (*MyD88*), *IKKγ*, TNFα induced protein 3 (*TNFAIP3*, also named *A20*), TNF receptor (TNFR)-associated factor 1 (*TRAF1*), *TRAF6*, etc. Thus, the percentage of genetic alterations from factors that can affect NF-κB signaling shall be much more than the 53.7% we count here. Therefore, the clinical data from these 348 CRC patients strongly suggest the pivotal role of genetic variations in both NF-κB family members and NF-κB pathway regulators may play in CRC initiation, progression, and prognosis [32].

Below, we will use PRMT5 as an example of a positive regulator, and ODAD2 as an example of a negative regulator to illustrate their genetic alterations in CRC in detail.

#### 3.2.2. Genetic Alterations of NF-κB Positive Regulator, PRMT5 in CRC

PRMT5, a type II PRMT enzyme, regulates gene expression, cell cycle, and protein function through arginine methylation [45]. PRMT5 is a critical player in various cancer types, including colorectal cancer, and its increased expression is common in these malignancies [34,41,42,43,46,47]. Overexpression in colorectal cancer suggests its role in oncogenesis via epigenetics and cell cycle, highlighting the potential for targeted therapy [43,45]. PRMT5 mutations are prevalent across a spectrum of cancer types, with a pronounced prevalence notably in CRC, pancreatic cancer, etc. This underscores their substantive role in the pathogenesis of these specific cancers, suggesting their potential as pivotal drivers [45]. Consequently, understanding the prevalence and significance of PRMT5 mutations holds promise for informing novel therapeutic interventions targeting the molecular underpinnings of skin and colorectal cancer. Notably, among the *PRMT1-9* genes, *PRMT5* gene is unique due to its higher occurrence of missense mutations. This suggests that changes in the coding sequence of *PRMT5* are more frequent in cancers, implying a potential role in cancer development [48]. Based on an analysis employing the Catalogue of Somatic Mutations in Cancer (COSMIC), a comprehensive collection indicates a total of 338 *PRMT5* mutations across various cancer types. Among these, 239 mutations are positioned in coding regions, while 99 are located in non-coding regions [48].

Ongoing extensive research is dedicated to deciphering the intricate mechanisms by which PRMT5 influences tumor formation, offering promising avenues for developing targeted and effective cancer treatments. PRMT5 has been previously shown to be overexpressed in approximately 75% of CRC patient tumor samples and negatively correlated with CRC patient survival. PRMT5 catalyzes the methylations of some proteins including NF-κB and Y-box binding protein 1 (YBX1), both are key transcriptional and translational regulators widely recognized as oncogenic drivers in various solid tumors, including CRC [49,50]. Our study has shown the impact of the Serine–Alanine mutant of PRMT5 (*S15A*) in HEK293 and CRC cells (HT29, DLD1, and HCT116) to understand its effect on NF-κB activation [49]. The results confirmed that S15 phosphorylation is crucial for PRMT5-mediated NF-κB activation. Overexpressing the S15A mutant significantly reduced NF-κB activation, indicating its inhibitory role. Moreover, the mutation disrupted the interaction between PRMT5 and p65 and led to decreased PRMT5 methyltransferase activity upon IL-1β stimulation.

**Figure 4:**
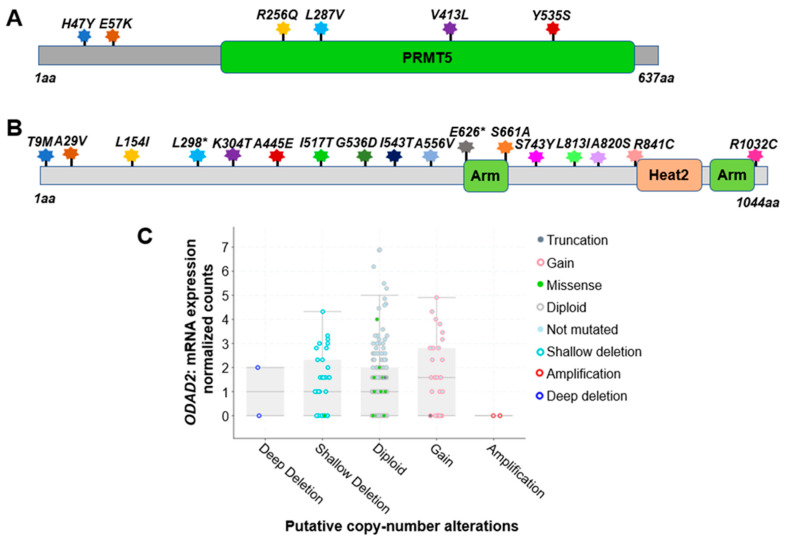
Genetic alterations on NF-κB signaling regulators PRMT5 and ODAD2. (**A**) Schematic diagram of protein mutations on PRMT5 identified in CRC patients. Data resource: cBioPortal for Cancer Genomics [31,32]. Note: PRMT5 is 637 amino acids (aa) in length. (**B**) Schematic diagram of protein mutations on ODAD2 identified in CRC patients. Data resource: cBioPortal for Cancer Genomics [31,32]. Note: ODAD2 is 1044aa in length. Note: Arm, Heat2 are domains on ODAD2. (**C**) Putative copy number alterations from GISTIC for ODAD2. Data resource: cBioPortal for Cancer Genomics [31,32]. Symbol *: Nonsense mutations.

Based on the analysis of 348 colon cancer patient samples using data from the CBioPortal [31,32], approximately 5.0% were found to harbor genetic alterations on the PRMT5 gene. Among these, six notable missense mutations were identified. They are *H47Y* (Histidine–Tyrosine); *E57K* (Glutamate–Lysine); *R256Q* (Arginine–Glutamine); *L287V* (Leucine–Valine); *V413L* (Valine–Leucine), and *Y535S* (Tyrosine–Serine) (Figure 4A). All of these are missense mutations. Additionally, there are cases of gene copy number gain.

This finding highlights the genetic heterogeneity within the studied population. The specific missense mutations at the residues listed above suggest potential functional implications that may contribute to the pathobiology of CRC. Further investigation into the consequences of this mutation and its role in tumorigenesis could offer valuable insights into the molecular mechanisms underlying CRC, potentially paving the way for targeted therapeutic approaches in personalized treatment strategies for affected patients [31,32].

In fact, PRMT5 currently is viewed as a hot therapeutic target in cancer. Several pharmaceutical companies are developing inhibitors to target this enzyme [42,43].

#### 3.2.3. Genetic Alterations of NF-κB Negative Regulator, ODAD2 in CRC

Recently, using the powerful validation-based insertional mutagenesis (VBIM) technique established by our laboratory previously [39,44], we discovered outer dynein arm docking complex subunit 2 (ODAD2) (also named armadillo repeat-containing protein 4 (ARMC4), a rarely studied protein known to date, as a novel negative regulator of NF-κB in CRC. We showed that a high expression of ODAD2 downregulated the expression of NF-κB-dependent genes, dramatically reduced NF-κB activity, cellular proliferation, anchorage-independent growth, and migratory ability in vitro, and significantly decreased xenograft tumor growth in vivo. Importantly, the lower ODAD2 expression in patient tumors than normal tissues indicate its potential tumor suppressor function in CRC. Collectively, we uncovered a completely new facet of ODAD2 function by identifying it as a novel NF-κB negative regulator, thus uncovering ODAD2 as a potential new therapeutic target in CRC [44].

Impressively, based on the analysis of 348 colon cancer patient samples using data from the CBioPortal [31,32], approximately 7.0% of patients were found to harbor genetic alterations of *ODAD2* (Table 3). Among these, 17 notable protein mutations were identified. For instance, mutations include L298* FS del (Leucine frame shift deletion), *A556V* (Alanine–Valine), and *A820S* (Alanine–Serine) missense mutation with ShallowDel (Shallow deletion), etc. (Figure 4B, Table 2). The types of gene alterations include missense mutation, deep deletion, amplification, etc. (Figure 4C). These data suggest that genetic alteration in *ODAD2* could be an important factor contributing to CRC initiation and progression.

## 4. Research Models CRC

As aforementioned, scientists have uncovered different genetic alterations along NF-κB signaling in CRC patients [31,32]. It would be of great importance if scientists could further utilize these patients’ samples to investigate their mechanistic roles in CRC initiation and progression, and build up a platform to develop novel drugs to treat CRC, and so on. The rapid advances in CRC research models in recent years have made all these applications possible. In the past decades, the field of CRC research has witnessed the emergence of models such as immortalized cancer cell lines, genetically engineered mouse models (GEMMs), cell line-derived xenografts (CDX), patient-derived xenografts (PDX), organoid cultures, and humanized mice, which have contributed distinct insights into the disease’s mechanisms, progression, and therapeutic treatments [51,52,53,54,55,56,57]. We have summarized the pros and cons of each model in Table 4. Among these models, PDX or PDX in humanized mice and organoid cultures, in particular, are emerging as more reliable preclinical CRC models. Thus, we will elaborate more detail on these two models below.

### 4.1. PDX CRC Models

**Figure 5:**
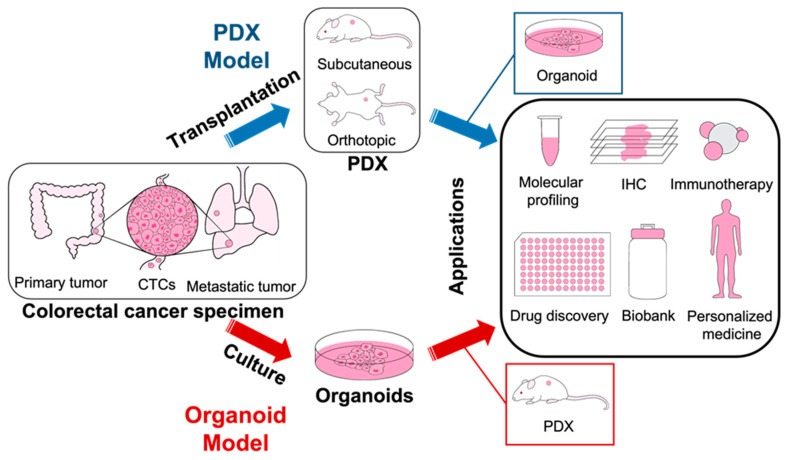
Generation and application of CRC PDX and organoid models. Both PDX and organoid models can be generated from primary tumors within the colon or rectum, metastatic tumors, or CTCs, which are surgically resected, biopsied, or collected via peripheral blood. For the PDX model (Top, blue arrows), surgically resected tumor tissues or biopsies will be transplanted into immunodeficient or humanized mice. The sliced tumor tissue or cell sample is transplanted either heterotopically through subcutaneous implantation into the dorsal area or orthotopically through direct implantation into the colon. When the tumor reaches sufficient size, it is dissected, confirmed, and re-passaged into other mice. For the organoid model (Bottom, red arrows), surgically resected tumor tissues or biopsies can be dissociated into single cells and then cultured in a 3D medium usually containing Matrigel and growth factors which allow and optimize growth. Both the PDX model and the organoids model can then be utilized by enabling molecular profiling of patient tumors, drug discovery, to facilitating personalized medicine. For the PDX model, it also can be further used to generate organoids. While for the organoid model, it also can be further used to generate the PDX model.

As shown in Figure 5 (Top Panel), PDX CRC models are generated by transplanting fresh primary tumor, metastatic tumor, or circulating tumor cells (CTCs) specimens into immunodeficient mice or humanized mice. It can be classified as subcutaneous and orthotopic PDX CRC model. For the subcutaneous PDX CRC model, it is completed heterotopically through subcutaneous implantation into the dorsal area; while for the orthotopic PDX CRC model, it is carried out orthotopically through direct implantation into the anatomical site of origin [52]. The subcutaneous PDX CRC model is commonly used as it is easy to operate, monitor, and resect with good tumor engraftment, while rarely generate metastases [66]. In contrast, the orthotopic PDX CRC model is more invasive, labor intensive, and difficult to monitor longitudinally; however, they are better models of metastases because they generate primary tumors and distant lung and liver metastases at similar rates observed in patients [64,66,67,68,69]. As such, orthotopic PDX CRC models are helpful to evaluate local invasive growth of primary tumors, study tumor–host interactions and therapeutic responses in their anatomical context, but preclinical therapeutic studies currently exclusively utilize subcutaneous PDX CRC models [70].

PDX CRC models faithfully recapitulate the stromal structure, histological differentiation, and histopathological subtypes of the patient tumors they are derived from [53,66,71]. In addition, studies have found that the PDX CRC models maintain important gene mutations (e.g., *KRAS*) as well as gene expression, copy number changes, and microsatellite instability of the primary tumor, enabling in vivo investigations mirroring human conditions [72]. As such, PDXs are suitable models for studying the NF-κB signaling pathway and have been utilized identify new biomarkers and novel targets. For instance, studies using PDX models have found that downregulation of RING finger 138 (RNF138) sensitized CRC cells to SC75741, a highly potent and specific NF-κB signaling inhibitor [73]. PDX models have also been used to identify NF-κB, EGFR, and 12 other markers of cetuximab sensitivity and resistance, and to elucidate the combined inhibition of NF-κB and BET proteins as a potential combination therapy for CRC [74,75]. Given that PDXs have been found to accurately predict patient responses to conventional as well as novel therapeutics, these models provide a valuable platform for investigating patient responses to drugs and treatments, thus elucidating the NF-κB signaling pathway in CRC, mechanisms of resistance, and facilitating personalized medicine [55,76] (Figure 5).

### 4.2. Organoid CRC Models

Different from PDX CRC models, organoid CRC models (Figure 5, Bottom Panel) are three-dimensional cultures that can be established from cancer cells from primary tumor samples, metastatic tumor samples, CTCs, or PDX CRC models (Figure 5). Organoid CRC models such as NF-κB reporter intestinal organoids can also be genetically modified (e.g., CRISPR-Cas9 genome editing) as well as generated from genetic modifying normal tissue organoids, enabling improved CRC visualization and carcinogenesis modeling [60,77,78,79,80]. Thus, their significance lies in their capacity to bridge the gap between traditional in vitro monolayer cultures and in vivo animal models. Since the first establishment of intestinal and human CRC organoids by Sato et al., organoid models have been utilized to elucidate various aspects of CRC such as intratumor heterogeneity, micrometastases, driver pathway mutations, and cancer stem cells [79,81,82,83]. These “tumor-in-a-dish” models have been found to accurately recapitulate the morphological and (epi)genetic features of parent tumors as well as the unique cellular and environmental characteristics that contribute to the vast biological inter- and intratumor heterogeneity [77,84,85]. Culture conditions can also be modified by adding or removing components (e.g., growth factors, cytokines) to investigate specific signaling pathways, enabling researchers to investigate unique TMEs and giving researchers additional in vitro control [86]. For instance, NF-κB reporter intestinal organoids are responsive to most cytokines such as TNFα, making them suitable models for investigating the role inflammatory factors, such as TNFα, play at different steps in the NF-κB pathway [87].

In addition, organoids have been found to recapitulate patient response [56,57]. As such, organoid models offer a valuable tool for modeling CRC in vitro due to their genetic modifiability, ability to recapitulate human CRC, and ability to predict patient responses to conventional and novel drugs and therapies [56,78,88] (Table 4, Figure 5).

### 4.3. Applications and Impact of PDX and Organoid CRC Models

Compared with CRC cells, GEMMs, and CDX models, PDX and organoid CRC models more accurately preserve patient tumor characteristics, represent cancer biology and heterogeneity, and recapitulate therapeutic responses. Hence, PDX and organoid models hold significant translational value and play complementary roles in CRC research.

PDX CRC models offer a complex in vivo environment by engrafting patient tumor tissues into immunodeficient mice, allowing the study of tumor behavior, therapy responses, and interactions with the TME. On the other hand, organoid CRC models provide an in vitro platform that captures cellular heterogeneity and maintains tumor architecture, enabling high-throughput drug screening, genetic manipulation, and detailed mechanistic studies. Utilized together, these models synergistically enhance translational research by providing both in vitro control and in vivo relevance, offering both preclinical and clinical insights into CRC biology and therapeutic responses [89,90].

The clinical relevance of PDX and organoid models also stem from their potential in immunotherapy research. Traditionally, it has been difficult to model and study tumor-immune system interactions and how immune cells react to drugs and therapeutics ex vivo. Through techniques such as adoptive cell-transfer therapy, tumor and immune cell co-cultures, and humanized mice, PDX and organoid models are increasingly being utilized to study CRC tumor-immune interactions as well as identifying immunotherapy targets in the NF-κB signaling pathway [65,91,92,93,94]. Humanized mice models, highly immunodeficient mice engrafted with functional human immune systems and PDXs or organoids, in particular, are emerging as models for developing and testing immunotherapeutic strategies and have provided many insights into the behaviors of diverse cancers within their native TMEs. As such, PDX and organoid models are improving to become more and more accurate in recapitulating CRC and tumor-immune system interactions, making both models valuable platforms for immunotherapy research as well as personalized medicine [93,94].

Moreover, the success rate to establish PDX and organoid CRC models is very high, which enables the prospective generation of large ”living biobanks” containing both collections of patients’ CRC samples, and their matched sets of PDX and organoid CRC models. Such “living biobanks” would offer not only unique insights into broad cancer phenotypes but also a platform for high-throughput drug screens, thus, facilitating a better understanding of CRC as well as the development of patient-specific treatment regimens [53,56,57] (Figure 5).

## 5. Conclusions and Perspective

Due to the fact that CRC is the third leading cause of cancer mortality in the United States [1], there is an urgent demand for the development of novel therapeutics for CRC. In addition to the standard therapy of CRC that we mentioned above, significant advances have also been made in the targeted therapy and immunotherapy fronts for CRC treatments. For example, several drugs targeting the EGFR, vascular endothelial growth factor receptor (VEGFR), and programmed cell death protein 1 (PD-1) have been approved by the FDA for CRC treatment. Some of these drugs include Cetuximab, Panitumumab, Ramucirumab, and Ipilimumab [4].

Due to the importance of NF-κB in CRC progression, agents that inhibit NF-κB can be developed as targeted therapies for CRC. Unsurprisingly, much effort has been made to develop NF-κB inhibitors [95,96]. However, targeting specific pathways that regulate NF-κB has been the preferred approach rather than targeting NF-κB itself, because basal NF-κB activity is vital to normal cellular functions and immune responses. To date, there are very few FDA-approved NF-κB inhibitors, and most focus on blood cancers; e.g., Bortizomib is a proteasome inhibitor not specific to NF-κB, and used on mantle cell lymphoma, and multiple myeloma, etc. Thus, there remains an urgent need to develop NF-κB inhibitors that target solid tumors, including CRC.

In this review, we provide detailed genetic alteration information about NF-κB signaling, including both NF-κB family members, and their regulators. This knowledge may serve as a rich reservoir for future study by the CRC scientific community. For instance, the function of each mutation could be individually tested in both in vitro and in vivo experimental models. A larger cohort of CRC patient specimens could be further collected to conduct much deeper and more complicated analysis, like the correlation between the mutation site and patients survival, CRC stage, gender, and race, etc.

On a broader perspective, since it is not surprising that CRC patients frequently harbor genetic mutations on more than one gene, the role of genetic alterations of NF-κB signaling components and their interaction with mutations on other genes, like KRAS or TP53 [8] (Figure 1) could be further delineated in CRC research models. In this regard, our review also contributes deep insight into different CRC research models, especially focusing on PDX and organoid models. Given the fact that genetic alterations on NF-κB signaling components account for at least nearly 53.7% of overall genetic changes identified in CRC patients [31,32], it is reasonably to argue that genetic alterations on NF-κB signaling may provide a fresh angle for novel therapeutic development. For instance, scientists may consider utilizing PDX or organoids to dive in the underlying mechanism of CRC, build up platforms for drug screening and development, and develop Biobanks, etc. Excitingly, the cutting-edge stem cell models, like isogenic human embryonic stem cell (hESC)-derived colonic cells and human pluripotent stem cells (hPSC)-derived colonic organoids can also be used to examine the role of specific gene mutations in CRC progression [97]. Collectively, these efforts may lead to the discovery of new therapeutic targets, and innovative personalized medicine for the treatment of CRC in the future.

## Figures and Tables

**Table 1:** Mutations observed on NF-κB components among 348 colon cancer patients (Data resource: cBioPortal for Cancer Genomics) [31,32].

Gene Symbol	Gene Description	Protein	Protein Mutation	Mutation Type	Copy Number
*Rel*	C-Rel proto-oncogene, NF-κB subunit	Rel	*R22C*	Missense	Diploid
			*G288S*	Missense	Diploid
			*R108Q*	Missense	Diploid
			*G229D*	Missense	Diploid
			*X285_splice*	Splice	Diploid
*RelA*	RelA proto-oncogene, NF-κB subunit	RelA (p65)	*R166W*	Missense	Diploid
			*D446H*	Missense	Diploid
			*N139del*	IF del	Diploid
			H487Pfs*4	FS ins	Diploid
			*P521L*	Missense	Diploid
			*T357A*	Missense	Diploid
			Q287*	Nonsense	ShallowDel
*RelB*	RELB Proto-Oncogene, NF-κB Subunit	RelB	*A29V*	Missense	Diploid
			*P314L*	Missense	Gain
			*R434W*	Missense	Diploid
			*T494M*	Missense	Diploid
			G530Afs*5	FS del	Diploid
			E53*	Nonsense	Diploid
			*P482L*	Missense	Diploid
			*P482L*	Missense	ShallowDel
			*V379I*	Missense	Diploid
			*G522R*	Missense	Diploid
			*Y539H*	Missense	Diploid
			*V353M*	Missense	Diploid
			*C306Y*	Missense	Diploid
*NF-κB1*	NF-κB subunit 1	p105/p50	*R613C*	Missense	Diploid
			*A901T*	Missense	Diploid
			*G477V*	Missense	Diploid
			*D436G*	Missense	Diploid
*NF-κB2*	NF-κB subunit 2	p100/p52	Y294Ifs*4	FS del	Diploid
			*A514T*	Missense	Diploid
			*A867V*	Missense	Diploid
			*K252N*	Missense	Diploid
			*T806M*	Missense	Diploid
			*L474M*	Missense	Diploid
			*A121T*	Missense	Diploid
			*K252M*	Missense	Diploid
			*M1?*	Nonstart	Diploid

FS ins: Frame shift insertion; FS del: Frame shift deletion; IF del: In frame deletion; ShallowDel: Shallow deletion; Symbol *: Nonsense mutations.

**Table 2:** Mutations observed on regulators of the NF-κB signaling pathway among 348 colon cancer patients (Data resource: cBioPortal for Cancer Genomics) [31,32].

Gene Symbol	Gene Description	Protein	Protein Mutation	Mutation Type	Copy Number
*Chuk* (*IKBKA*)	Inhibitor of nuclear factor κB kinase subunit α	IKKα	E82*	Nonsense	Diploid
			*P700del*	IF del	Diploid
			*X577_splice*	Splice	Diploid
			E513*	Nonsense	Diploid
			*L50P*	Missense	ShallowDel
*IKBKB*	Inhibitor of nuclear factor κB kinase subunit β	IKKβ	R582Q	Missense	Diploid
			*P551L*	Missense	Diploid
			*A454T*	Missense	Diploid
			N225Tfs*25	FS del	Gain
			*Q438H*	Missense	Gain
			*A481V*	Missense	Diploid
			*X159_splice*	Splice	Gain
*IRAK1*	Interleukin 1 receptor associated kinase 1	IRAK1	*R51C*	Missense	Diploid
			*T383A*	Missense	Diploid
			*T234M*	Missense	Diploid
			*A78T*	Missense	Diploid
			*R61C*	Missense	Diploid
			*E259D*	Missense	Diploid
			*C43R*	Missense	Diploid
*KDM2A*	Lysine demethylase 2A	KDM2A	P597Afs*34	FS ins	Diploid
			*T162M*	Missense	Diploid
			*N1083S*	Missense	Diploid
			*H452R*	Missense	Diploid
			*A576S*	Missense	Diploid
			*P729L*	Missense	Diploid
			*R733G*	Missense	Diploid
			*S416G*	Missense	Diploid
*MAP3K7*	Mitogen-activated protein kinase kinase kinase 7	MAP3K7 (TAK1)	*R226W*	Missense	Diploid
			T169Dfs*7	FS ins	Diploid
			*R238Q*	Missense	Diploid
			*L255V*	Missense	Diploid
			*D488V*	Missense	Diploid
			*R248Q*	Missense	Diploid
			*P256S*	Missense	Diploid
			*D343Y*	Missense	Diploid
			*R463K*	Missense	Diploid
*NFKBIA*	NF-κB inhibitor α	IκBα	*E41A*	Missense	Diploid
			*P147H*	Missense	Diploid
*ODAD2*	Outer dynein arm docking complex subunit 2	ODAD2	*T9M*	Missense	Diploid
			*R841C*	Missense	Diploid
			*R1032C*	Missense	Diploid
			*K304T*	Missense	Diploid
			*I517T*	Missense	Diploid
			*L813I*	Missense	Diploid
			*A29V*	Missense	Diploid
			*A556V*	Missense	Diploid
			L298*	FS del	Gain
			E626*	Nonsense	Diploid
			*I543T*	Missense	Diploid
			*A445E*	Missense	Diploid
			*L154I*	Missense	Diploid
			*G536D*	Missense	Diploid
			*A820S*	Missense	ShallowDel
			*S661A*	Missense	Diploid
			*S743Y*	Missense	Diploid
*PRMT5*	Protein arginine methyltransferase 5	PRMT5	*H47Y*	Missense	Diploid
			*V413L*	Missense	Diploid
			*R256Q*	Missense	Diploid
			*Y535S*	Missense	Diploid
			*L287V*	Missense	Diploid
			*E57K*	Missense	Gain
*TAB1*	TGFβ activated kinase 1 (MAP3K7) binding protein 1	TAB1	*Y293C*	Missense	Diploid
			*E96D*	Missense	Diploid
			*A310G*	Missense	Diploid
			*L361Q*	Missense	Diploid
*TAB2*	TGF-β activated kinase 1 (MAP3K7) binding protein 2	TAB2	*A672T*	Missense	Diploid
			*R579I*	Missense	Diploid
			*N211K*	Missense	Diploid
			*R72C*	Missense	Diploid
			*A182V*	Missense	Diploid
*TRAF2*	TNF receptor associated factor 2	TRAF2	P9Lfs*77	FS del	Diploid
			P9Lfs*77	FS del	Amp
			*E122K*	Missense	Diploid
			*A3T*	Missense	Diploid
			*A494V*	Missense	Diploid

Amp: Amplification; FS ins: Frame shift insertion; FS del: Frame shift deletion; IF del: In frame deletion; ShallowDel: Shallow deletion; Symbol *: Nonsense mutations.

**Table 3:** Protein Mutation Rate of NF-κB family members or representative NF-κB pathway regulators among 348 CRC patients (Data resource: cBioPortal for Cancer Genomics) [31,32].

Classification	Protein	Protein Mutation Types	Alteration Frequency, %	Total Protein Mutation Types		Total Alteration Frequency, %	
NF-κB family members	Rel	5	2.5	38	113	14.8	53.7
	RelA (p65)	7	2.5				
	RelB	13	5.0				
	P105/p50	4	1.8				
	P100/p52	9	3.0				
NF-κB pathway regulators	IKKα	5	1.4	75		38.9	
	IKKβ	7	6.0				
	IRAK1	7	4.0				
	KDM2A	8	2.8				
	MAP3K7	9	4.0				
	NFKBIA	2	1.1				
	ODAD2	17	7.0				
	PRMT5	6	5.0				
	TAB1	4	1.8				
	TAB2	5	3.0				
	TRAF2	5	2.8				

**Table 4:** Advantages and disadvantages of different CRC models.

Model	Advantages	Disadvantages	Reference
Immortalized cancer cell lines	Practical, inexpensive Large quantity, permit amplification, experimental replication, and storage	Accumulate genetic aberrations Lack invasiveness, metastasis, tumor heterogeneity, and TME	[58,59]
GEMMs	Genetically defined Evaluate the functions of specific genes and molecular pathways of tumorigenesis Mimic TSI	Lack invasiveness, metastasis, and tumor heterogeneity Time consuming, expensive	[51,52,58]
CDXs	Cell lines are generally available and modifiable Fast, inexpensive High take rates	Do not reflect histopathological traits of human tumors Lack invasiveness, metastasis, tumor heterogeneity, and TME Fail to evaluate the immune system	[53,59,60]
PDXs	Retain original tumor histological and genetic traits Model metastatic behavior RTR Easy to expand	Varied take rates Fail to evaluate the immune system Time consuming, expensive	[54,55,61]
Organoids	Retain original tumor histological and genetic traits RTR Genetically engineerable to reflect mutations and for visualization	Varied take rates Complex culture components Lack TME	[56,57,62,63]
Humanized mice	Mimic human immunological response and TME RTR Creates a natural heterogeneity of tumor cells	Cross-reaction between human factors and mouse cells can reduce engraftment Technically complicated, expensive	[53,64,65]

RTR: Recapitulate therapeutic response; TME: Tumor microenvironment; TSI: Tumor–stroma interactions.
